# Clinical characteristics associated with somatic *GNAS* mutations in acromegaly: a systematic review and institutional experience

**DOI:** 10.3389/fendo.2026.1736208

**Published:** 2026-01-21

**Authors:** Brendan R. Dillon, Margaret Ruddy, Emily C. McQuade, Shruti N. Shah, Alberta Twi-Yeboah, Benjamin A. Levinson, Nidhi Agrawal

**Affiliations:** 1 Holman Division of Endocrinology, Diabetes and Metabolism, Department of Medicine, New York University Langone Health, New York, NY, United States; 2 Department of Medicine, New York University Langone Health, New York, NY, United States; 3 New York University Grossman School of Medicine, New York, NY, United States; 4 Division of Biostatistics, Department of Population Health, New York University Langone Health, New York, NY, United States

**Keywords:** acromegaly, clinical characteristics, GNAS, postoperative outcomes, somatotroph tumors

## Abstract

**Introduction:**

Acromegaly is a rare, insidious disease associated with significant morbidity and mortality usually caused by a growth hormone (GH)-secreting pituitary tumor. Somatic mutations in *GNAS* are common in these tumors, yet their diagnostic, prognostic, and therapeutic implications are less clear.

**Methods:**

We conducted a structured review of the literature and meta-analysis to investigate the association of *GNAS* mutation status with clinical characteristics and treatment outcomes in adult patients with acromegaly. This was complemented by an analysis comparing patients with acromegaly and identified tumor somatic *GNAS* mutations versus those without at our affiliated institution, NYU Langone Health.

**Results:**

We identified 55 publications that met our inclusion criteria, all observational in nature and most retrospective in design. Twenty-two patients with acromegaly at our institution underwent pituitary tumor resection followed by tumor somatic mutation analysis from 2022 to 2024. The aggregate prevalence of somatic *GNAS* mutations in acromegaly was 38% in the systematic review, which was similar to the prevalence of 41% at our institution. While some studies in our review found patients with *GNAS* mutated tumors were older and more frequently male, most did not find this association. Whether these tumors demonstrate greater GH secretory capacity is unclear. There was greater consistency in findings that *GNAS*+ tumors are smaller and possibly less invasive. While greater GH suppression to acute octreotide treatment was frequently reported in patients with *GNAS*+ tumors, most studies that investigated the response to long-term somatostatin receptor ligand (SRL) therapy did not find an association between *GNAS* mutation presence and biochemical control. At our institution, patients with *GNAS*+ tumors were older at the time of surgery and most classified as mammosomatotroph adenomas on pathology.

**Conclusions:**

Despite their high prevalence, *GNAS* mutations cannot reliably inform prognosis and treatment in acromegaly based on findings to date. Larger and prospective studies are needed exploring the frequency and intensity of preoperative symptoms and comorbidities, postoperative outcomes, and occurrence of prolactin co-secretion in *GNAS*+ tumors.

**Systematic Review Registration:**

https://www.crd.york.ac.uk/prospero/, identifier CRD420251107763.

## 1 Introduction

Acromegaly is a rare, chronic condition marked by growth hormone (GH) hypersecretion most often due to a pituitary somatotroph adenoma with a wide range of clinical effects including physical, cardiovascular, pulmonary, metabolic, musculoskeletal, oncologic, and neuropsychiatric (1–3). Diagnostic delay occurs in a majority of patients with a reported mean duration exceeding five years contributing to increased morbidity and mortality (4). While transsphenoidal resection is first-line therapy, surgical remission is achieved in only approximately 50% depending on tumor size and invasiveness as well as surgical center expertise (1, 5, 6). In the setting of persistent disease, medical therapy is needed targeting the somatotroph adenoma or antagonizing GH peripherally with the goal of achieving biochemical control (1, 3, 7), which is associated with normalization of mortality (8). Even when such control is achieved, symptoms and impaired quality of life frequently persist (9–12). Ongoing follow up is required for all patients to monitor disease status and address comorbidities and complications (2, 3, 7).

Greater understanding of the molecular mechanisms underlying pituitary tumor pathogenesis has the potential to better inform prognosis and treatment planning (13). Somatic, gain-of-function mutations in *GNAS* encoding the stimulatory G-protein alpha subunit (Gsα) are well-established molecular drivers in acromegaly with a prevalence of approximately 40% (14). These missense point mutations occur at codon 201 or 227 in a heterogenous pattern and impair the intrinsic, inhibitory GTPase activity of Gsα resulting in constitutive activation of the cyclic adenosine monophosphate (cAMP) pathway necessary for GH secretion and cell proliferation (15, 16). Studies investigating the clinical characteristics of *GNAS* mutations in acromegaly have yielded inconsistent results, although there is suggestion these tumors may be associated with older age at diagnosis, male sex, smaller size yet enhanced GH secretory ability, less invasion, densely granulated cytokeratin pattern, and greater response to somatostatin receptor ligand (SRL) therapy (17–33).

A better understanding of the diagnostic and therapeutic implications of somatic *GNAS* mutations in acromegaly is needed. Our goals were to describe the clinical features of pituitary somatotroph tumors harboring these mutations through a systematic review of the literature with meta-analysis supplemented by an analysis comparing patients with acromegaly with and without tumor somatic *GNAS* mutations at our institution.

## 2 Methods

### 2.1 Study protocol

This systematic review was conducted according to PRISMA 2020 statement guidelines and registered on PROSPERO (ID CRD420251107763). Our objectives were to investigate the association between *GNAS* mutation status and clinical features as well as treatment outcomes in adults with acromegaly.

### 2.2 Eligibility criteria

Studies were considered eligible for inclusion if they were published in a peer-reviewed journal, were written in English or with available English translation, and included adult patients with acromegaly and identified somatic *GNAS* mutations on surgical pathology molecular testing. Given the nature of the exposure, nonrandomized study types were included. Animal studies, studies with pediatric patients, case reports, reviews, book chapters, and conference proceedings were excluded.

### 2.3 Search strategy

**Figure 1:**
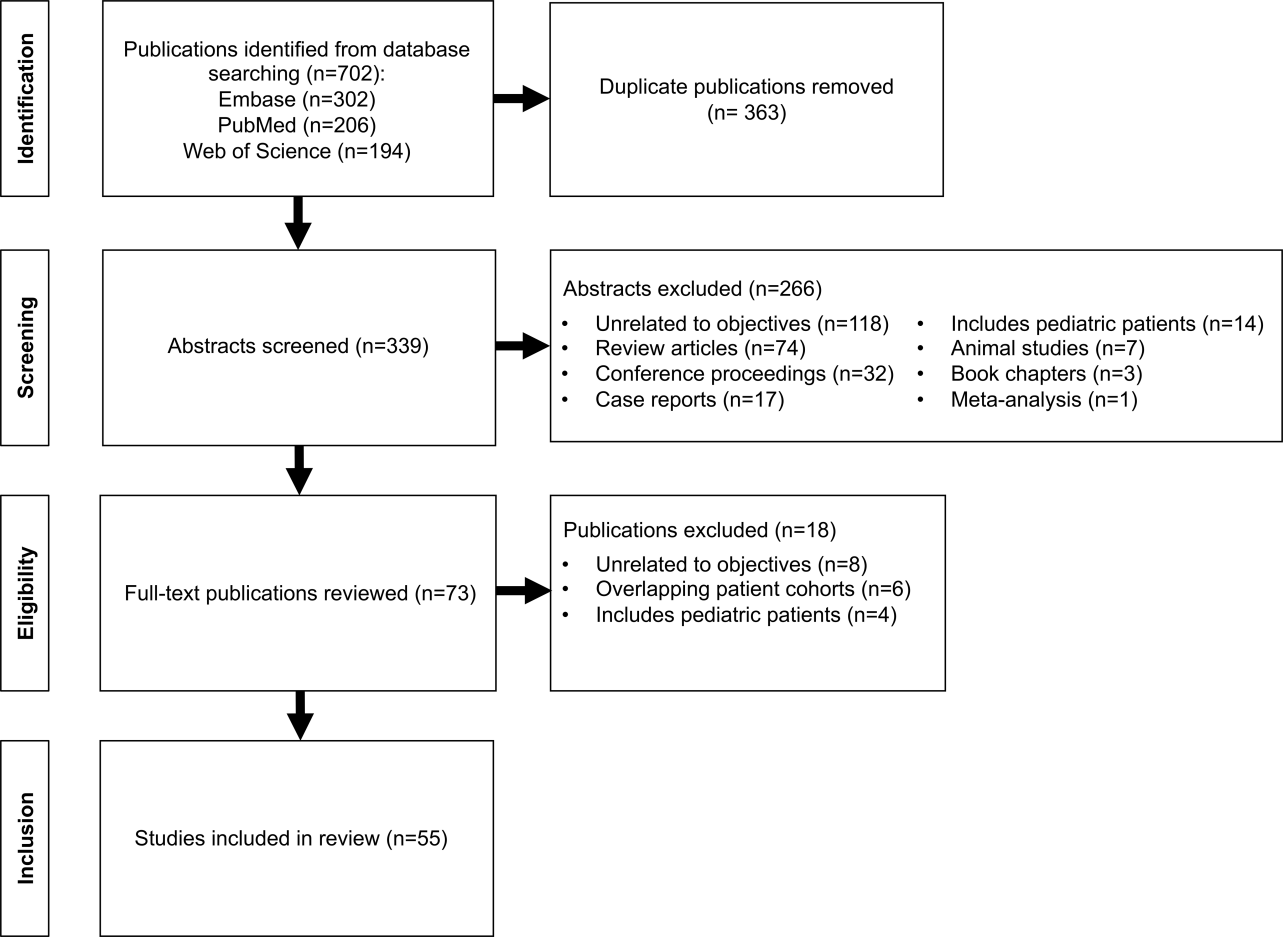
Flow diagram for article selection.

Searches were conducted through Embase, PubMed, and Web of Science databases with no date restrictions. The following search terms were used in the search strategies: (“acromegaly” OR “acromegalies” OR “acromegalic” OR “growth hormone-secreting pituitary adenoma” OR “growth hormone-secreting tumor” OR “somatotroph adenoma” OR “pituitary tumor” OR “growth hormone-secreting” OR “growth hormone-producing” OR “growth hormone hypersecretion” OR “GH-secreting” OR “GH-producing” OR “GH hypersecretion”) AND (“GNAS” OR “stimulatory G-protein alpha subunit” OR “Gs protein” OR “Gs alpha subunit” OR “G protein alpha subunit” OR “Gs alpha gene” OR “Gs alpha mutation” OR “G protein mutation” OR “Gsp” OR “Gps”). **Figure 1** illustrates the article selection process. After removal of duplicate publications, abstract screening, and review of the full-text for selected publications to assess for eligibility, 55 studies were included in the review. For two studies, data from two separate patient cohorts were reported, which we maintained in our data collection and analysis.

### 2.4 Data extraction

Four independent investigators screened the abstracts identified in the initial literature searches for relevance. Next, the full text of each selected article was reviewed to evaluate for inclusion. If two studies had overlapping patient cohorts, only one was included and was selected based on either a more complete reporting of clinical characteristics or a larger cohort size if both reported equally on clinical characteristics. Data extraction was completed by four independent investigators using Microsoft Excel. Variables of interest included demographics, symptoms, comorbidities, radiological features, pathological features, pre- and post-surgical hormone levels, and postsurgical outcomes. Age at diagnosis and age at surgery were considered equivalent for purposes of analysis. Hormone level values were converted to ng/mL for purposes of analysis.

### 2.5 NYU institutional experience

In the second component of this study, we investigated the clinical characteristics of adult patients with acromegaly with and without somatic *GNAS* mutations at our affiliated institution, NYU Langone Health in New York, NY. With assistance from the Department of Pathology Neuropathology Service, we identified all somatotroph adenomas that were resected and underwent genetic testing for somatic mutations from 2022 to 2024 following the advent of NYU Langone Genome PACT (Profiling of Actionable Cancer Targets) testing at our institution. NYU Langone Genome PACT is a diagnostic, qualitative, *in vitro* targeted next generation sequencing tumor profiling test that detects tumor gene alterations in a 607-gene panel. The electronic medical records of these patients were retrospectively reviewed and data collected related to the same variables of interest as the systematic review component. This component of the study was approved by the NYU Langone Health Institutional Review Board (protocol i24-00566) and determined to be exempt from full informed consent due to minimal risk.

### 2.6 Statistical analysis

SPSS software and R language software were used for statistical analysis.

#### 2.6.1 Meta-analysis

A mixed-effects meta-analysis was conducted to estimate the overall mean for continuous variables and the overall proportion for binary variables across studies. The random-effects model accounted for between-study heterogeneity by allowing the true effect sizes to vary across studies.

For continuous variables, the fixed effect was the study-specific mean, and the random effect was study.

If the mean was missing, we applied the following approximation methods for the mean in the order of priority, depending on the availability of other summary statistics, including median, minimum, maximum, first quartile (Q1), and third quartile (Q3):

1. Method A (34):


$$\text{Mean}\approx \frac{\text{min}+2*\text{median}+\text{max}}{4}\;$$

For small samples (*n* < 25)

Method B (35, 36):


$$\text{Mean}\approx \frac{\text{Q}1+\text{median}+\text{Q}3}{3}$$

2. Method C:

Mean ≈ Median

General statistical practice, used as fallback

If the standard deviation (SD) was missing, we used the following approximation methods in the order of priority, depending on the availability of other parameters, including median, minimum, maximum, Q1, Q3, and interquartile range (IQR):

Method A (34):


$$\text{SD}\approx \surd \left(\frac{{\left(\text{min}-\text{median}\right)}^{2}+{\left(\text{max}-\text{median}\right)}^{2}+{\left(\text{max}-\text{min}\right)}^{2}}{3}\right)$$

1. Method B (34):


$$\text{SD}\approx \frac{\text{max}-\text{min}}{4}$$

For samples with *n* ≤ 70

Method C (34, 35):


$$\text{SD}\approx \frac{\text{max}-\text{min}}{6}$$

For samples with *n* ≥ 70

Method D (35, 36):


$$\text{SD}\approx \frac{\text{Q}3-\text{Q}1}{1.35}\;$$

Classical normal distribution property; popularized in meta-analysis literature

Method E (35, 36):


$$\text{SD}\approx \frac{\text{IQR}}{1.35}$$

Classical normal distribution property; popularized in meta-analysis literature

For binary variables, proportions were transformed using the logit function to map values from the bounded [0, 1] scale to the entire real line, stabilizing variances and meeting model assumptions of normality and homoscedasticity. Specifically, the logit transformation was applied as:


$$\text{logit}\left(p\right)=\log\left(\frac{p}{1-p}\right)$$

where *p* is the observed proportion.

Meta-analyses and mixed-effects modeling were then performed on the logit-transformed scale.

For interpretation and presentation, all model estimates and confidence intervals (CIs) were back-transformed to the original proportion scale using the inverse logit transformation:


$$p=\frac{e^{\text{logit}\left(p\right)}}{1+e^{\text{logit}\left(p\right)}}$$

This approach allowed effect estimates and heterogeneity measures to be reported as proportions with corresponding 95% CIs.

#### 2.6.2 NYU institutional experience

Descriptive statistics were calculated for the *GNAS*+ and *GNAS*- groups for each variable. Data were expressed as mean ±SD for continuous variables and *n* (%) for categorical variables. Continuous variables were compared using Student’s *t*-test or Wilcoxon-Mann-Whitney test. Categorical variables were compared using Fisher’s exact test. A *p* value< 0.05 was considered significant.

## 3 Results

### 3.1 Types of studies

This systematic review included 55 studies with a total of 57 patient cohorts due to the two studies that reported two separate patient cohorts each. All studies were observational. The majority were retrospective (*n* = 41), while 14 studies were prospective in design. The cohorts were stratified by size as follows with most having 50 or fewer patients: *n* ≤ 25 (20 cohorts), *n* = 26 – 50 (20 cohorts), *n* = 51 – 75 (10 cohorts), *n* = 76 – 100 (3 cohorts), and *n* ≥ 100 (4 cohorts). Year of publication ranged from 1990 to 2024. The geographic location of studies included Asia, Australia, Europe, North America, and South America.

### 3.2 Prevalence of *GNAS* mutations

The prevalence of somatic *GNAS* mutations was recorded for each patient cohort (**Table 1**). The total number of patients with acromegaly was 2, 540. The prevalence of *GNAS* mutations in acromegaly ranged from 4 – 63% with an aggregate prevalence of 38%. Methods for somatic genetic testing varied among cohorts and included PCR-DNA direct sequencing analysis (*k* = 44), RT-PCR direct sequencing analysis (*k* = 5), PCR and oligonucleotide-specific hybridization (*k* = 3), PCR-single-strand conformation polymorphism analysis followed by sequencing (*k* = 2), targeted capture sequencing (*k* = 1), multiplexed next-generation sequencing (*k* = 1), and whole-exome sequencing (*k* = 1). For 847 patients with acromegaly and *GNAS*+ tumors, the location of the mutation was specified and occurred most commonly at codon Arg201 (84%) with the remaining occurring at codon Gln227. For 669 of these tumors, the specific mutation was specified with the following reported frequencies: Arg201Cys (75%), Gln227Leu (13%), Arg201His (6%), Arg201Ser (3%), Gln227Arg (3%), and Gln227Glu (<0.01%). In two cases, mutations in both Arg201 and Gln227 were present. In one study, three previously unreported *GNAS* mutations (Gly49Arg, Ser111Asn, Ala249Asp) were found in the somatotroph adenoma from one patient (32). Most studies did not include testing for other somatic or germline gene mutations. In one cohort with 26 *GNAS*+ tumors, no somatic mutations in *GHR* were found (25). In one case, the presence of *GNAS* mutation was associated with allelic deletions of chromosome 11 (37). Among three cohorts with a total of 101 patients with *GNAS*+ tumors, co-occurring missense somatic mutations in *AIP* were detected in 4% (28, 32, 38). Among two cohorts with a total of 74 patients with *GNAS*+ tumors, no co-occurring somatic mutations in *PRKACA* were found (32, 39). In one study with five *GNAS*+ tumors, no *USP8* somatic mutations were detected but polymorphisms in *USP8* at exon 1 and the 14-3–3 binding domain were found in three cases (39). In one study involving targeted capture sequencing of 36 genes, among 69 *GNAS*+ tumors, there were three identified missense mutations in *USP8*, one missense mutation in *GPR101*, two missense mutations in *PRKACB*, one missense mutation in *PRKAR1A*, one missense mutation in *ARRB1*, two frameshift mutations in *ARRB2*, two missense mutations in *SSTR5*, and four missense mutations in *MEN1* (32). Finally, in one study with whole-exome sequencing, among 11 *GNAS*+ tumors, there was co-occurrence of mutations in other genes involved in cAMP signaling in two (39).

**Table 1:** Prevalence of *GNAS* mutations in somatotroph adenomas in included studies.

Country	Year	*n*	Prevalence (%)	Country	Year	*n*	Prevalence (%)
Australia	1995 (84)	13	31	Germany, Italy	2016 (39)	31	16
Brazil	2009 (45)	54	15		2016 (39)	36	31
	2011 (46)	39	10	Japan	1993 (47)	45	4
	2018 (38)	41	27		1993 (48)	43	9
	2021 (31)	136	40		2003 (60)	20	55
	2022 (49)	74	40		2006 (61)	100	53
Canada	1992 (85)	15	40		2008 (62)	20	55
China	1998 (40)	40	55		2009 (63)	56	46
	2000 (55)	18	33		2010 (43)	43	58
	2010 (64)	43	33		2014 (41)	67	39
	2019 (29)	25	28		2016 (27)	61	51
	2024 (33)	97	44		2022 (32)	121	57
	2024 (69)	44	36	Mexico	2005 (22)	58	19
Finland	2017 (28)	59	36	Norway	2012 (44)	74	49
France	1995 (65)	15	27	Poland	2022 (68)	134	39
	1998 (19)	30	27		2024 (86)	48	17
	2020 (67)	53	25	South Korea	1996 (21)	21	43
	2021 (87)	23	39		2001 (20)	44	16
	2021 (87)	82	23		2004 (70)	16	63
Germany	1993 (18)	19	42		2021 (30)	126	60
	2013 (26)	28	36	Spain	2020 (42)	50	33
Italy	1990 (88)	18	44	Turkey	2003 (89)	7	43
	1998 (90)	26	42	USA	1990 (17)	25	40
	1998 (58)	18	36		1992 (56)	26	35
	2001 (78)	19	37		1995 (57)	30	33
	2001 (59)	21	38		2007 (23)	60	40
	2003 (66)	8	38		2017 (91)	21	29
Belgium, France, Italy	2013 (24)	39	36	UK	1993 (37)	11	18
					2013 (25)	49	53

*n* total number of somatotroph adenomas with genetic testing for *GNAS* mutations.

### 3.3 Clinical characteristics of *GNAS* mutations in acromegaly

A comparison of *GNAS*+ versus *GNAS*- tumors with respect to clinical characteristics was reported in 38 studies. The reported associations of *GNAS* mutation with sex, age, basal hormone levels, tumor size, tumor invasion, tumor proliferation, postsurgical remission, and GH suppression with oral glucose tolerance testing (OGTT) and SRL treatment preoperatively and postoperatively are shown in **Table 2**. The overall means for continuous variables and overall proportions for binary variables for *GNAS*+ tumors across studies are displayed in **Table 3**.

**Table 2:** Association of *GNAS* mutation status with preoperative and postoperative clinical characteristics as reported by included studies.

Reference	*n*, total tested for *GNAS* mutation	*n*, *GNAS+*	Association of *GNAS* mutation with preoperative and postoperative clinical characteristics												
			Sex	Age	Basal GH	Basal IGF-1	Basal PRL	Tumor size	Tumor invasion	Pre-op OGTT response	Pre-op SRL response	Ki-67	Post-op OGTT response	Postsurgical remission	Post-op SRL response
(17)	25	10	No	No	No^a^	--	No^b^	Smaller	No^c^	Greater	--	--	--	--	--
(56)	26	9	No	No	No	No	Higher	No	No	--	--	--	--	--	--
(18)	19	8	M>F^d^	No	Lower^e^	No	No	No	More	Greater	--	No	Greater	--	--
(57)	30	10	No	No	No	--	No	No	--	--	--	--	--	--	Greater^f^
(21)	21	9	No	Older	No	--	No	No	--	No	Greater^g^	--	--	--	--
(19)	30	8	No	No	No^h^	No	Higher	No	Less^e^	--	Greater	--	--	No	Greater
(58)	18	8	--	--	No	--	--	No	--	--	--	--	--	--	--
(40)	40	22	No	No	No	--	No	No	No	Greater	--	--	Greater	Higher	--
(55)	18	6	No	Older^e^	Higher^e^	--	No	Smaller^e^	No	--	--	--	--	--	--
(20)	44	7	No	No	No	--	No	Smaller	No	No	No	--	--	No	--
(59)	21	8	--	--	No	No	--	Smaller	--	--	--	--	--	--	--
(60)	20	11	--	No	No	No	--	No	--	--	--	--	--	--	--
(70)	16	10	--	--	--	--	--	--	--	--	Greater	--	--	--	--
(22)	58	11	No	No	Lower^e^	No	No	No^i^	--	--	--	--	No	No	--
(61)	100	53	No	No	No	No	--	--	--	--	No	--	--	--	--
(23)	60	24	No	No	Higher^e^	Higher	--	Smaller^e^	No	--	--	No	No	No	No
(62)	20	11	No	No	No	No	--	No	--	--	--	--	--	--	--
(45)	54	8	No	No	Higher^e^	Higher^e^	--	Larger^e^	--	--	--	--	--	--	--
(63)	56	30	--	--	No	No	--	--	--	--	Greater^e^	No	--	--	--
(43)	43	25	No	No	No^j^	--	--	Smaller	--	--	Greater^e^	No	--	--	--
(64)	43	14	No	No	No	No	No	No	No	--	--	--	--	--	--
(46)	39	4	--	--	--	--	--	--	--	--	No	--	--	--	--
Reference	*n*, total tested for *GNAS* mutation	*n*, *GNAS+*	Association of *GNAS* mutation with preoperative and postoperative clinical characteristics												
			Sex	Age	Basal GH	Basal IGF-1	Basal PRL	Tumor size	Tumor invasion	Pre-op OGTT response	Pre-op SRL response	Ki-67	Post-op OGTT response	Postsurgical remission	Post-op SRL response
(44)	74	36	--	--	--	--	--	No	--	--	No	--	--	--	No^k^
(25)	49	26	M>F^l^	No	No	No	No	No	No	--	Greater	No	--	--	--
(24)	39	14	No	No	No	No	No	No	No	--	Greater^e^	No^m^	--	--	--
(41)	67	26	No	No	No	No	--	No	No	--	Greater	--	--	--	--
(27)	61	31	No	No	No^n^	No	No	Smaller	No	No	Greater	No	--	--	--
(28)	59	21	No	No	No	No	Higher^e^	No	Less^e^	--	--	No	--	--	--
(38)	41	11	No	No	No	No	--	No	No	--	--	No	--	No	No
(29)	25	7	No	No	Higher	Higher	--	Smaller	Less	--	--	Lower	--	--	--
(67)	53	13	No	No	--	--	--	No	No	--	--	--	--	--	--
(42)	50	--	--	No	--	--	--	No	--	--	--	--	--	--	No
(31)	136	54	No	No	No	No	--	Smaller	Less	--	--	--	--	--	No
(30)	126	75	M>F°	No	No	Higher	--	No	No	No	--	No	Greater^p^	Higher	--
(68)	134	52	No	No	--	--	--	No	No	--	--	--	--	--	--
(32)	121	69	No	No	Higher^e^	No	Higher	Smaller	Less	--	Greater^q^	--	No	No	--
(33)	97	43	M>F^r^	No	Higher^e,j^	Higher^e^	--	Smaller	No	No	--	Lower	No	No	--
(69)	44	16	No	No	--	--	--	--	Less	--	--	--	No	No	No

GH growth hormone, IGF-1 insulin-like growth factor 1, PRL prolactin, OGTT oral glucose tolerance test, SRL somatostatin receptor ligand.

--Not reported.

a GH level was lower in *GNAS*+, but there was no difference when controlled for tumor size.

b No difference when controlled for tumor size.

c Tumor invasion was greater in *GNAS*-, but there was no difference when controlled for tumor size.

d The prevalence of *GNAS* mutations was 67% in male patients and 18% in female patients.

e Trend reported but not statistically significant.

f Effect assessed *in vitro* via cell culture from surgical tissue.

g Of the 21 patients, 19 had no previous treatment for acromegaly, one had surgery 7 years prior, and one had radiation 5 years prior.

h When GH level per tumor diameter ratio considered, greater secretory activity noted in *GNAS*+.

i Microadenomas were more frequent in *GNAS*+, but this was not statistically significant.

j When GH level per tumor volume ratio considered, greater GH-producing index noted in *GNAS*+.

k No difference in GH reduction with SRL treatment was noted, but greater tumor reduction was seen in *GNAS*+.

l The prevalence of *GNAS* mutations was 70% in male patients and 37% in female patients.

m No difference in Ki-67 for untreated somatotroph adenomas. Ki-67 lower in *GNAS*+ tumors treated preoperatively with SRL therapy compared to treated *GNAS*- tumors.

n *GNAS*+ tumors with higher GH levels corresponding to tumor size.

°The prevalence of *GNAS* mutations was 76% in male patients and 48% in female patients.

p *GNAS*+ tumors with lower nadir GH on immediate postoperative OGTT, but no difference noted on postoperative OGTT at 6 months.

q GH decrease by octreotide test greater in *GNAS*+ tumors but not significant, while GH decrease by preoperative SRL therapy significantly greater in *GNAS*+ tumors.

r The prevalence of *GNAS* mutations was 58% in male patients and 33% in female patients.

**Table 3:** Meta-analysis of the clinical characteristics of *GNAS*+ mutations in acromegaly.

Continuous variables								
Variable	Number of studies contributed	Estimated overall mean	Lower 95% CI	Upper 95% CI	*p*-value	Significant at 5%	Study random effect variance	Heterogeneity score
Age (years)	35	45.9	44.4	47.4	< 0.0001	Yes	11.7	64.6
Basal GH (ng/mL)	34	31.3	25.1	37.6	< 0.0001	Yes	165.1	83.6
Basal IGF-1 (ng/mL)	17	824.7	707.7	941.8	< 0.0001	Yes	45517.3	97.9
Basal IGF-1 (xULN)	5	2.7	1.5	3.8	0.003	Yes	0.6	71.5
Basal PRL (ng/mL)	12	23.7	15.5	31.8	< 0.0001	Yes	99.4	79.7
Tumor volume (cm^3^)	7	1.6	1.0	2.3	0.0008	Yes	0.3	69.9
Tumor largest dimension (cm)	21	1.7	1.5	1.9	< 0.0001	Yes	0.1	82.7
Ki-67 (%)	7	1.3	0.4	2.2	0.01	Yes	0.9	98.2
Postoperative GH (ng/mL)	3	3.4	0	25.2	0.57	No	50.4	0
Postoperative IGF-1 (ng/mL)	3	250	18.4	481.7	0.04	Yes	5581.7	95.8
Binary variables								
Variable	Number of studies contributed	Estimated overall proportion	Lower 95% CI	Upper 95% CI	*p*-value	Significant at 5%	Study random effect variance	Heterogeneity score
Female sex (%)	35	51.4	48.1	54.8	0.38	No	0	0
Macroadenoma (%)	16	78.3	69.4	85.1	< 0.0001	Yes	0.2	36.3
Cavernous sinus invasion (%)	14	25.2	15.1	38.8	0.003	Yes	0.8	71.6
Densely granulated (%)	5	56.2	31.3	78.4	0.54	No	0.5	78.1
Sparsely granulated (%)	5	36.2	15.3	64.1	0.24	No	0.7	83.6
Mixed pattern granulation (%)	2	12.8	1.4x10^-5^	100	0.33	No	1.8	74.9
Surgical remission (%)	7	51.9	29.2	73.8	0.85	No	0.8	76.9
Remission at last follow up (%)	4	53.2	29.6	75.4	0.71	No	0.2	33.3

CI, confidence interval; GH, growth hormone; IGF-1, insulin-like growth factor 1; PRL, prolactin; ULN, upper limit of normal.

#### 3.3.1 Demographics, preoperative symptoms and comorbidities, preoperative hormone levels

Only a limited number of studies (*n* = 8) explicitly reported on preoperative symptoms and signs, comorbidities, or disease duration. Most did not find a difference between patients with and without *GNAS* mutations (18, 20, 22, 27, 40–42), although one study reported increased diagnostic delay and frequency of vision impairment without increased visual field deficits in patients with *GNAS* mutations (33).

With respect to sex, 30 studies reported the association with *GNAS* mutation status. *GNAS*+ tumors were more common in male patients in four of these studies with the remainder finding no association. Using data from 35 studies, the estimated overall proportion female was 51.4% for patients with *GNAS*+ tumors (95% CI [48.1 – 54.8%], *p* = 0.38, *I*^2^ = 0%).

With respect to age, 32 studies reported the association with *GNAS* mutation status with all but one finding no significant difference. Using data from 35 studies, the estimated overall mean age was 45.9 years for patients with *GNAS*+ tumors (95% CI [44.4 – 47.4 years], *p* < 0.0001, *I*^2^ = 65%).

In 31 studies, the association between *GNAS* mutation status and basal GH level was reported with most finding no association. While six found a trend of higher GH level with *GNAS*+ tumors, the difference was only significant in one study (29). However, four studies did note a higher secretory capacity in *GNAS*+ tumors when accounting for tumor size (19, 27, 33, 43). Using data from 34 studies, the estimated overall mean basal GH level was 31.3 ng/mL for patients with *GNAS*+ tumors (95% CI [25.1 – 37.6 ng/mL], *p* < 0.0001, *I*^2^ = 84%). In 23 studies, the association between *GNAS* mutation status and basal insulin-like growth factor 1 (IGF-1) level was reported with most finding no association. Five studies reported a higher IGF-1 level with *GNAS*+ tumors with this difference being significant in three. Patients with *GNAS*+ tumors had an estimated overall mean basal IGF-1 level of 824.7 ng/mL (95% CI [707.7 – 941.8 ng/mL], *p* < 0.0001, *I*^2^ = 99%) using data from 17 studies and an estimated mean IGF-1 level expressed as a multiple of the upper limit of normal (ULN) of 2.7 (95% CI [1.5 – 3.8], *p* = 0.003, *I*^2^ = 72%) using data from five studies. Fewer studies (*n* = 16) reported the association between *GNAS* mutation status and basal prolactin level with 13 finding no association and three finding significantly higher prolactin levels with *GNAS*+ tumors. Using data from 12 studies, the overall mean basal prolactin level was 23.7 ng/mL (95% CI [15.5 – 31.8 ng/mL], *p* < 0.0001, *I*^2^ = 80%) for patients with GNAS+ tumors. With respect to GH reduction with OGTT, a greater response was demonstrated in patients with *GNAS*+ tumors in three out of eight studies. Preoperative response to SRL therapy either by acute octreotide testing or more sustained treatment was assessed in 14 studies with most finding a trend of greater response in the *GNAS*+ group, which was significant in seven studies.

#### 3.3.2 Radiological features

The association between *GNAS* mutation status and tumor size was reported in 33 studies. When compared to *GNAS*- tumors, *GNAS*+ tumors were significantly smaller in nine studies. Tumors with *GNAS*+ tumors had an estimated overall mean tumor volume of 1.6 cm^3^ (95% CI [1.0 - 2.3 cm^3^], *p* = 0.0008, *I*^2^ = 70%) using data from seven studies and an estimated overall mean tumor diameter of 1.7 cm (95% CI [1.5 - 1.9 cm], *p* < 0.0001, *I*^2^ = 83%) using data from 21 studies. Using data from 16 studies, the estimated overall proportion of *GNAS*+ tumors classified as macroadenomas was 78.3% (95% CI [69.4 – 85.1%], *p* < 0.0001, *I*^2^ = 36%). The association between *GNAS* mutation status and tumor invasion was reported in 23 studies with significantly less invasion in 4, more invasion in 1, and no difference in 18. Using data from 14 studies, the estimated overall proportion of *GNAS*+ tumors with cavernous sinus invasion as assessed on preoperative magnetic resonance imaging (MRI) was 25.2% (95% CI [15.1 - 38.8%], *p* = 0.003, *I*^2^ = 72%).

#### 3.3.3 Pathological features

The association between *GNAS* mutation status and tumor proliferation as assessed by Ki-67 proliferation index was reported by 12 studies with lower Ki-67 reported in two studies and no association in the remainder. Using data from seven studies, the estimated overall mean Ki67 of *GNAS*+ tumors was 1.3% (95% CI [0.4 – 2.2%], *p* = 0.01, *I*^2^ = 98%). Using data from three studies, the estimated overall proportion with Ki67< 3% was 74.4% (95% CI [60.2 – 84.7%], *p* < 0.02, *I*^2^ = 0%).

Granulation pattern data was reported in six studies. *GNAS* mutations were more common in densely granulated adenomas compared to sparsely granulated adenomas in one study (26), but no significant difference in granulation pattern between *GNAS*+ and *GNAS*- was reported in four studies (25, 31, 41, 44). Using data from five studies, in *GNAS*+ tumors, the estimated overall proportion of densely granulated adenomas was 56.2% (95% CI [31.3 – 78.4%], *p* = 0.54, *I*^2^ = 78%), sparsely granulated adenomas was 36.2% (95% CI [15.3 – 64.1%], *p* = 0.24, *I*^2^ = 84%), and mixed pattern adenomas was 12.8% (95% CI [1.4x10^-5^ – 100%], *p* = 0.33, *I*^2^ = 75%).

#### 3.3.4 Postoperative outcomes

Compared to preoperative data, data on postoperative outcomes was comparatively less reported, and there was greater heterogeneity with respect to measures used and timing. The association between *GNAS* mutation status and postsurgical remission was reported in 10 studies with greater remission for *GNAS*+ tumors found in two. Using data from seven studies, the estimated overall proportion of patients with *GNAS*+ tumors with postsurgical remission was 51.9% (95% CI [29.2 – 73.8%], *p* = 0.85, *I*^2^ = 77%). Using data from three studies, patients with *GNAS*+ tumors had an estimated overall mean postoperative GH level of 3.4 ng/mL (95% CI [0 – 25.2 ng/mL], *p* = 0.57, *I*^2^ = 0%) and mean postoperative IGF-1 level of 250.0 ng/mL (95% CI [18.4 – 481.7 ng/mL], *p* = 0.04, *I*^2^ = 96%).

### 3.4 NYU institutional experience

A total of 22 patients with acromegaly underwent transsphenoidal resection at NYU Langone Health followed by tumor somatic mutation genetic testing from 2022 to 2024. Somatic, activating *GNAS* mutations were identified in 9 out of 22 patients (41%). The identified mutations were Arg201Cys in five patients and Gln227Leu in four patients. The clinical characteristics grouped by *GNAS* mutation status are displayed in **Table 4**. Patients with *GNAS*+ tumors were older at the time of surgery compared to patients with *GNAS*- tumors with respective mean ages of 59.6 and 39.2 years (*p* = 0.003). There were no significant differences in preoperative symptoms or comorbidities in patients with *GNAS*+ versus *GNAS*- tumors. Preoperative GH, IGF-1, and prolactin levels were higher in patients with *GNAS*+ tumors, but these differences were not statistically significant. Additionally, tumor volume and largest dimension did not differ. Postoperative GH level was lower in patients with *GNAS*+ tumors (mean 2.7 versus 3.9 ng/mL, *p* = 0.01). Postoperative prolactin level was also lower (mean 4.7 versus 10.3 ng/mL, *p* = 0.006). Postoperative IGF-1 was lower in the *GNAS*+ group but this difference was not significant. Similarly, postoperative decreases in GH and IGF-1 were greater in the *GNAS*+ group but not significant. With respect to pathological diagnosis, seven out of nine *GNAS*+ tumors demonstrated dual GH and prolactin staining with six mammosomatotroph adenomas (MSA) and one mixed somatotroph-lactotroph adenoma (MSLA). Tumor DNA methylation profiling revealed a class of pituitary adenoma, STH densely granulated, group B in all *GNAS*+ tumors. Molecular testing demonstrated *NTRK3*-*SH3GL3* gene fusion in one *GNAS*+ tumor. No other somatic tumor mutations were found. Germline genetic testing was obtained in four patients with *GNAS*+ tumors and two patients with *GNAS*- tumors. Heterozygous mutations in *CHEK2* and *APC* were found in one patient with a *GNAS*- tumor. No pathogenic germline variants were detected in the other five patients.

**Table 4:** Clinical characteristics of patients with acromegaly with and without tumor somatic *GNAS* mutations.

Clinical characteristics and postsurgical outcomes	*GNAS*+ (*n* = 9)	*GNAS*- (*n* = 13)	*p*-value
Sex (Male/Female)	4/5	7/6	NS
Age at surgery (years)	59.6 ± 14.0	39.2 ± 10.7	0.003
Headache	33%	69%	NS
Acromegalic facial features	89%	85%	NS
Arthralgias	78%	54%	NS
Dysglycemia	67%	54%	NS
Hyperlipidemia	78%	62%	NS
Hypertension	67%	54%	NS
Obstructive sleep apnea	56%	38%	NS
Carpal tunnel syndrome	33%	23%	NS
Thyroid nodule(s)	78%	46%	NS
Colon polyp(s)	56%	46%	NS
Visual problems	33%	23%	NS
Visual field deficit	0%	8%	NS
Abnormal periods	11%	8%	NS
Sexual dysfunction	11%	23%	NS
Preoperative GH (ng/mL)	34.3 ± 29.2	22.6 ± 40.5, 1 ND	0.08
Preoperative IGF-1 (xULN)	2.8 ± 0.7	2.3 ± 0.7, 1 ND	NS
Preoperative PRL (ng/mL)	34.3 ± 33.1	20.4 ± 15.2, 1 ND	NS
Tumor volume (cm^3^)	2.8 ± 2.4	4.5 ± 7.3	NS
Tumor largest dimension (cm)	2.0 ± 0.5	2.0 ± 0.9	NS
Cavernous sinus invasion	44%	46%	NS
Suprasellar extension	44%	46%	NS
Bone invasion	11%	8%	NS
Optic chiasm compression	11%	15%	NS
MRI T2 hyperintensity	22%	8%	NS
Preoperative SRL therapy	22%	0%	NS
Ki-67< 3%	78%	54%	NS
Pathological diagnosis PSA MSA MSLA Plurihormonal	22% 67% 11% 0%	46% 8% 8% 38%	0.01
Methylation class PITAD STH DNS A PITAD STH DNS B PITAD STH SPA	0% 100% 0%	54% 8% 38%	<0.0001
Postoperative day 1 GH (ng/mL)	1.2 ± 0.9, 1 ND	5.3 ± 11.6, 2 ND	NS
Postoperative GH (ng/mL)	2.7 ± 1.5	4.0 ± 11.4, 1 ND	0.01
Postoperative GH delta (ng/mL)	-31.6 ± 28.8	-18.6 +/- 29.4	NS
Postoperative IGF-1 (xULN)	0.9 ± 0.3	1.2 ± 0.8, 2 ND	NS
Postoperative IGF-1 delta (ng/mL)	-1647.1 ± 3059.2, 1 ND	-393.5 ± 218.1, 3 ND	0.07
Postoperative PRL (ng/mL)	4.7 ± 1.8, 1 ND	10.3 ± 7.1, 2 ND	0.006
Postsurgical remission	57%, 2 ND	64%, 2 ND	NS
Postoperative hypogonadism	50%, 3 ND	44%, 4 ND	NS
Postoperative hypothyroidism	13%, 1 ND	18%, 2 ND	NS
Postoperative adrenal insufficiency	22%	25%, 1 ND	NS
Postoperative hypopituitarism	11%	0%, 1 ND	NS
Postoperative permanent AVP deficiency	0%	8%	NS
Postoperative radiotherapy	0%	8%	NS
Postoperative SRL therapy	22%	31%	NS

AVP arginine vasopressin, GH, growth hormone; IGF-1, insulin-like growth factor 1; MRI, magnetic resonance imaging; MSA, mammosomatotroph adenoma; MSLA, mixed somatotroph-lactotroph adenoma; ND, patients with missing data; NS, not significant; PITAD STH DNS A, pituitary adenoma STH densely granulated group A; PITAD STH DNS B, pituitary adenoma STH densely granulated group B; PITAD STH SPA, pituitary adenoma STH sparsely granulated; PRL, prolactin; PSA, pure somatotroph adenoma; SRL, somatostatin receptor ligand; ULN, upper limit of normal.

## 4 Discussion

We investigated the clinical characteristics of patients with acromegaly due to somatotroph adenomas harboring somatic, activating *GNAS* mutations through a structured review of the literature and comparison of patients with and without *GNAS* mutations at our institution.

Our findings confirm current understanding regarding the prevalence of *GNAS* mutations in acromegaly with an aggregate prevalence of 38% from the systematic review and 41% at our institution. In this systematic review, the included patient cohorts spanned five continents. With respect to differences by country, some earlier Brazilian and Japanese studies reported lower prevalence rates (45–48); however, larger and more recent studies are not suggestive of a difference between these two countries and others (27, 31, 32, 41, 49). Somatic, activating *GNAS* mutations in somatotroph adenomas have been characterized as heterozygous, missense mutations occurring most frequently at Arg201 and less frequently at Gln227, which are most often identified via targeted gene sequencing (14). Whole genome and exome sequencing studies of GH-secreting adenomas have demonstrated *GNAS* mutation prevalence rates of 25 – 54% (39, 50–53). Regarding the variability in the reported prevalence rates of *GNAS* mutations in acromegaly among the included studies in this systematic review which span a time period over 30 years, it is likely that use of different sequencing technologies and evolution in techniques over time partly account for this variation. Additionally, a majority of the studies focused their sequencing of *GNAS* at codons 201 and 227 within exons 8 and 9, respectively, and did not include testing for other somatic tumor mutations. Somatic variants in genes encoding G protein-coupled receptors (GPCRs) and proteins involved in cAMP or calcium signaling in patients with acromegaly with *GNAS*+ tumors have been identified (32, 39). Future investigation is needed to better understand the role that novel *GNAS* variants and co-existing variants in other genes may play in the pathogenesis of acromegaly. For instance, emerging data have identified unique genetic variants that may influence the tumor microenvironment through immune cell activation and infiltration and thereby contribute to differences in disease phenotype (54).

While the prevalence of somatic *GNAS* mutations in acromegaly is relatively well established, the diagnostic, prognostic, and therapeutic implications are less certain. In this systematic review, a limited number of studies reported data for preoperative symptoms and signs, comorbidities, or disease duration, and most found no difference between patients with and without *GNAS*+ tumors (18, 20, 22, 27, 40–42). However, one study found *GNAS*+ tumors were associated with a significantly longer diagnostic delay (33). Unlike most studies in the review, we found patients with *GNAS*+ tumors were significantly older at the time of surgery than patients with *GNAS*- tumors in our institution’s cohort. It is possible diagnostic delay led to this finding, although data regarding occurrence of first comorbidity and time of diagnosis were not recorded. Additionally, we found no difference in the frequencies of symptoms, signs, and comorbidities in patients with and without *GNAS* tumor mutations. Similar to most studies in the review, we found no association between *GNAS* mutation status and sex.

It has been suggested that somatotroph adenomas with *GNAS* mutations are smaller yet demonstrate enhanced GH secretion related to constitutive activation of the cAMP pathway normally induced by stimulation of the growth hormone-releasing hormone (GHRH) receptor (29, 32, 33, 43). In our review, while some studies reported a trend of higher GH levels in patients with *GNAS*+ tumors (23, 32, 45, 55), most reported no association (17, 20, 21, 24, 25, 28, 30, 31, 38, 40, 41, 56–64) with only one reporting a significantly higher GH level (29), only four reporting significantly higher GH levels when accounting for tumor size (19, 27, 33, 43), and two reporting a trend of lower GH levels (18, 22). With respect to IGF-1 levels in patients with *GNAS*+ tumors compared to those with *GNAS*- tumors, most reported similar levels (18, 19, 22, 24, 25, 27, 28, 31, 32, 38, 41, 56, 59–64) with three reporting significantly higher levels (23, 29, 30) and two reporting only a trend of higher levels (33, 45). In our institution’s cohort, GH and IGF-1 levels were higher in patients with *GNAS*+ tumors, but these differences were not significant. Activation of the cAMP pathway with its downstream effects, such as phosphorylation of transcription factor cAMP response element-binding protein (CREB), is considered a key component in somatotroph tumorigenesis regardless of *GNAS* mutation status (53, 65, 66). Lower expression of *PDE4D* encoding a phosphodiesterase (PDE) enzyme involved in hydrolysis and deactivation of cAMP was demonstrated in somatotroph adenomas compared to gonadotroph and lactotroph adenomas (53). However, *GNAS*+ and *GNAS*- tumors do not necessarily differ in cAMP levels which has been attributed to significantly greater PDE activity in mutated tumors (58). Thus, while *GNAS* mutations may result in greater GH secretion, increased PDE activity and other factors may attenuate this effect resulting in no difference in GH levels.

Regarding tumor size, several studies including those with some of the largest sample sizes found *GNAS*+ tumors were significantly smaller than *GNAS*- tumors (17, 20, 27, 29, 31–33, 43, 59), although many found no association (18, 19, 21, 22, 24, 25, 28, 30, 38, 40–42, 44, 56–58, 60, 62, 64, 67, 68). In one study, combination of data with earlier series led to a finding of significantly smaller size among *GNAS*+ tumors with suspicion that earlier studies were likely limited in their ability to find an effect due to small sample sizes (23). In our cohort, *GNAS*+ tumor volume was lower but not significantly different with the ability to find an effect possibly limited by our relatively small sample size. With respect to tumor invasion, most initial studies did not find an association with *GNAS* mutation status (20, 23, 40, 55, 56, 64), while some more recent studies found *GNAS*+ tumors were less invasive (29, 31, 32, 51, 69). In our cohort, there was no difference in cavernous sinus invasion with respect to *GNAS* mutation status. Demonstration of increased expression of cell-cycle checkpoints p53 and p21^Wif1/Cip1^ in somatotroph adenomas led to the hypothesis that this finding may contribute to restricted growth and expansion in *GNAS*+ tumors (53). Additionally, increased expression of the large non-coding RNA and tumor suppressor *MEG3* in *GNAS*+ GH-secreting tumors has been implicated in limiting invasion via inactivation of the Wnt/β-catenin signaling pathway (29, 69). If *GNAS*+ tumors are associated with reduced tumor proliferation, Ki67 index is an inconsistent marker of this based on the studies in our review, as several studies found no association (18, 23, 25, 27, 28, 30, 38, 43, 63), and two found Ki67 indices were lower in mutated tumors (29, 33). In our cohort, a greater proportion of *GNAS*+ tumors demonstrated Ki67 indices less than 3%, but the difference was not significant. If *GNAS*+ tumors are smaller and less invasive, then postsurgical remission may be higher in this subset of tumors. In the limited number of studies reporting postsurgical data, most did not find a difference (19, 20, 22, 23, 32, 33, 38, 69). In the two studies reporting higher postsurgical remission in *GNAS*+ tumors, the two groups did not differ in terms of tumor size and invasion (30, 40), which suggests other factors modulate this possible association. In our small cohort, rates of surgical remission did not differ among patients with versus without *GNAS* mutations. Future investigation with greater standardization of postoperative measures is needed to confirm if prognostic differences exist with respect to surgical resection.

At a molecular level, SRL therapy targets the cAMP pathway which is constitutively activated in somatotroph adenomas with *GNAS* mutations (53). Several studies have investigated the association of *GNAS* mutation status and SRL treatment response with both acute and long-term administration (19–21, 23–25, 27, 31, 32, 38, 41–44, 46, 61, 63, 69, 70). Across these studies, findings are inconsistent regarding whether *GNAS* mutations are associated with a more favorable response. A meta-analysis of eight studies with 310 patients who underwent acute octreotide suppression testing demonstrated more pronounced GH suppression in patients with *GNAS*+ tumors (weighted mean difference 9.08%, 95% CI [2.73-15.42], *p* = 0.005) (71). However, response to acute octreotide treatment does not necessarily portend long-term efficacy in acromegaly (7, 72–74). Most studies that have evaluated postoperative biochemical control with SRL therapy in the setting of residual disease have found no difference related to the presence of a somatic *GNAS* mutation (23, 31, 38, 42, 44, 69). The findings of our review support the recently published consensus statement from the 15^th^ Acromegaly Consensus Conference with discretionary recommendation against using the presence of *GNAS* mutations to predict SRL treatment response and guide selection of medical therapy either preoperatively or following surgical resection with inadequate disease control (7). However, greater quality evidence through larger and prospective studies would be beneficial. IGF-1 dynamics during standard-dose and high-dose or high-frequency first-generation SRL therapy can predict long-term biochemical response and inform therapeutic decisions (75). Future investigation should explore the effect of *GNAS* mutation status, if any, in predicting response to different modes of SRL therapy as assessed by IGF-1 dynamics.

Densely granulated cytokeratin pattern and high somatostatin receptor 2 (SSTR2) expression are recommended factors for predicting postoperative SRL response (7). In this review, most studies that evaluated granulation pattern did not find a difference between tumors with and without *GNAS* mutations (25, 31, 41, 44), while in one study, *GNAS* mutations were more common in densely granulated adenomas (26). Findings with respect to SSTR2 expression in *GNAS*+ tumors are mixed with some studies reporting higher expression (46, 76, 77) and others reporting no difference (31, 70, 78, 79). In one series, the presence of *GNAS* mutation was associated with increased frequency of plasma-membrane-dominant staining of SSTR2A (63). However, while GH reduction with acute octreotide administration was greater in the patients with *GNAS*+ tumors, this difference did not reach statistical significance. Interestingly, somatostatin receptor 5 (SSTR5) expression was found to be lower in *GNAS*+ tumors in a large cohort study (31). Further investigation is needed with respect to postoperative outcomes and SRL response in tumors with *GNAS*+ tumors in association with granulation pattern and SSTR2/5 expression.

A notable finding in our cohort of patients with acromegaly is the high proportion of *GNAS*+ tumors with dual GH- and prolactin-staining immunohistochemistry (IHC) with the majority being classified as mammosomatotroph adenomas. While *GNAS* mutations occur in GH and prolactin co-secreting adenomas, prior studies have not found a significant difference in prolactin staining or histological diagnosis between tumors with and without *GNAS* mutations (19, 43, 56, 68), and one found less prolactin staining in the *GNAS*+ group (21). With regards to possible hypersecretion, higher basal prolactin levels have been found in some cohorts of *GNAS*+ tumors (19, 28, 32, 56). In our cohort, preoperative prolactin levels were higher but not significantly different in patients with *GNAS*+ tumors. Approximately 25% of GH-secreting adenomas demonstrate prolactin staining (80). Proper histological classification of these tumors may have prognostic implications, as MSAs have been associated with smaller size, less invasion, and greater total gross resection, while MSLAs the inverse with greater hyperprolactinemia (81–83). Future investigation should explore the prevalence of *GNAS* mutations in dual GH- and prolactin-staining tumors and the association with clinical features, including those associated with prolactin hypersecretion, and postoperative outcomes.

Our findings must be interpreted in the context of our study’s limitations. First, all of the studies included in our review are observational in nature and most retrospective in design, thereby increasing the risk of bias and confounding factors. Most study samples were small to moderate in size, which limits generalizability due to possible selection bias and the ability to detect effects. For example, the introduction of somatic tumor mutation testing at our institution allowed for retrospective analysis of patients with and without *GNAS* mutations, but incorporation of this testing into clinical care is not uniform and there may be unique factors related to the decision to pursue analysis, such as preoperative features, radiographic evidence of invasiveness, and intraoperative findings. Additionally, the results of our meta-analysis for the clinical characteristics of *GNAS*+ tumors are limited by the variation in and evolution of hormone assays over time as well as variation in the units used requiring conversion. Furthermore, there was variability with respect to the methods and timing of postoperative assessment as well as proposed criteria for biochemical control and remission. Finally, interpretation of the pooled outcomes from the meta-analysis is limited when heterogeneity is high.

In conclusion, our systematic review and institutional experience confirms the prevalence of somatic *GNAS* mutations in acromegaly. At our institution, we found patients with *GNAS*+ tumors were significantly older, had lower postoperative growth hormone and prolactin levels, and were most likely to have a tumor pathology diagnosis of dual GH- and prolactin-staining adenoma. These tumors may be smaller yet demonstrate enhanced secretory ability with less invasion. Additional investigation is needed to better understand how these mutations are associated with the frequency and intensity of preoperative symptoms and comorbidities as well as postoperative outcomes. It is unclear if *GNAS* mutations predict long-term SRL treatment efficacy in the setting of residual disease. The possible association between *GNAS* mutation and GH and prolactin co-secreting adenomas should be explored.

## Data Availability

The raw data supporting the conclusions of this article will be made available by the authors, without undue reservation.
